# Urine-based point-of-care detection of direct oral anticoagulant activity in acute stroke—accuracy at anti-Xa thresholds relevant for intravenous thrombolysis

**DOI:** 10.1016/j.rpth.2025.103331

**Published:** 2025-12-29

**Authors:** Stefan T. Gerner, Alina Braemer, Martin B. Juenemann, Anne Mrochen, Tobias Braun, Linus Olbricht, Norma J. Diel, Ulrich J. Sachs, Hagen B. Huttner, Omar Alhaj Omar

**Affiliations:** 1 Department of Neurology, University Hospital Giessen, Germany; 2 Justus-Liebig University (JLU) Giessen, Germany; 3 Department of Neurology, University Hospital Erlangen, Germany; 4 Center for Transfusionmedicine and Hemotherapy, University Hospital Giessen, Germany

**Keywords:** direct oral anticoagulants, factor-Xa-inhibitors, intracerebral hemorrhage, oral anticoagulation, point-of-care testing, thrombin-inhibitors

## Abstract

**Background:**

Rapid assessment of direct oral anticoagulant (DOAC) activity is essential in acute ischemic stroke (AIS), particularly because intravenous thrombolysis (IVT) may be considered at anti-Xa levels ≤ 100 ng/mL. Laboratory drug-specific assays; however, are often limited by availability and turnaround time. A urine-based point-of-care (POC) test may provide a rapid alternative.

**Objectives:**

To determine the diagnostic accuracy of a urine-based POC dipstick for detecting clinically relevant DOAC activity at (1) the established screening threshold (≥30 ng/mL) and (2) the IVT-relevant threshold (≥100 ng/mL), using calibrated plasma DOAC levels as a reference standard.

**Methods:**

In this prospective diagnostic accuracy study (UPTURN trial), consecutive AIS patients underwent urine-based POC testing. Dipstick results, recorded as visual and automatic readouts, were analyzed against plasma DOAC activity. Relevant anticoagulant activity was defined as anti-Xa ≥30 ng/mL; the IVT eligibility threshold was defined as anti-Xa <100 ng/mL. Factor Xa inhibitors were quantified using drug-specific chromogenic anti-Xa assays; dabigatran activity was measured using Biophen DTI. Diagnostic accuracy metrics were calculated with exact 95% CIs. Time-to-result was compared between POC testing and plasma assays.

**Results:**

Among 101 AIS patients (55 with DOAC intake), the urine-based dipstick test reliably identified relevant anticoagulatory activity (anti-Xa ≥30 ng/mL) with a sensitivity of 100% and specificity of 96.3% (automated readout). Visual interpretation yielded similar accuracy. For higher anti-Xa levels (≥100 ng/mL), sensitivity remained 100%, though specificity decreased (74.4%). A double-positive visual result increased specificity to 92.8% at 84.4% sensitivity. Median time to result was 19 minutes for urine testing versus 144 minutes for blood-based assays. Test performance was consistent across DOAC agents, dosages, and intake timing. Visual grading enabled semiquantitative discrimination of DOAC levels.

**Conclusion:**

Urine-based DOAC dipstick testing is a rapid, accurate, and reliable method for detecting anticoagulatory activity in AIS patients, providing a valuable tool to guide acute therapeutic decisions. Future studies are warranted to validate its clinical impact and broader applicability, especially in the emergency setting.

## Essentials

• Many stroke patients use direct anticoagulants, complicating timely intravenous thrombolysis decisions.
• We evaluated a urine dipstick in acute stroke care to assess factor Xa-inhibitor activity.
• The dipstick rapidly identified patients with relevant anticoagulatory activity, indicating intravenous thrombolysis unsuitability.
• Fast exclusion of relevant activity via urine dipstick may support safe and timely thrombolysis.

## 1 Introduction

Direct oral anticoagulants (DOACs) have revolutionized anticoagulation therapy, offering advantages such as predictable pharmacokinetics and reduced monitoring requirements compared to traditional vitamin K antagonists (VKAs) [[1], [2], [3], [4]]. However, their increasing use has introduced new challenges in acute ischemic stroke (AIS) management, particularly in patients eligible for intravenous thrombolysis [[5], [6], [7], [8]]. International guidelines contraindicate thrombolysis in the presence of therapeutic anticoagulation due to the heightened risk of hemorrhagic complications [9,10]. Identifying patients with DOAC-related anticoagulatory activity below critical thresholds is crucial to safely extending thrombolysis to this population [11,12].

Current observational evidence suggests that, in patients receiving factor Xa inhibitors (FXaIs), anti-Xa activity ≤100 ng/mL may permit consideration of intravenous thrombolysis (IVT) after careful evaluation of bleeding risk [13,14]. This IVT-relevant threshold complements the traditionally used ≥30 ng/mL laboratory benchmark, which has long served as a conservative indicator of clinically relevant anticoagulatory activity in pharmacological and diagnostic studies. Reliable assessment of these thresholds is therefore of high clinical relevance for decision-making in AIS. Further, blood-based testing methods, such as chromogenic assays, often require centralized laboratory facilities, which are not widely available and contribute to delays that are incompatible with the time-sensitive nature of thrombolysis [15,16].

A urine-based point-of-care (POC) DOAC dipstick has recently emerged as a rapid and technically simple method to detect the presence of DOACs [17,18]. he test includes separate pads for FXaIs and dabigatran and provides qualitative or semiquantitative readouts within minutes. Early feasibility studies have demonstrated promising sensitivity and specificity, yet data in well-characterized stroke populations—particularly analyses aligned with clinically relevant DOAC thresholds—remain limited [19].

Against this background, we conducted a prospective diagnostic accuracy study in consecutive AIS patients to evaluate the performance of a urine-based POC test compared with calibrated plasma DOAC measurements. Specifically, we aimed to determine the test’s ability to (i) detect relevant anticoagulant activity at the established ≥30 ng/mL benchmark and (ii) assess discriminatory performance at the IVT-relevant threshold (<100 ng/mL). Furthermore, we compared time-to-result between POC testing and plasma assays to quantify potential workflow advantages.

## 2 Methods

### 2.1 Study design and setting

We conducted a investigator driven prospective, single-center diagnostic accuracy study (UPTURN trial) in consecutive patients presenting with acute ischemic stroke (AIS) to a certified stroke center of a German university hospital. The study protocol was approved by the institutional ethics committee and registered on ClinicalTrials.gov (Identifier: NCT06037200). Written informed consent was obtained from all participants or their legal representatives, in accordance with the principles of the Declaration of Helsinki. The first 44 patients included in this study (23 DOAC-treated and 21 controls) were part of an earlier feasibility pilot published as Doeppner et al. [19]. These patients represented the initial enrollment phase of the same ongoing prospective registry and were therefore included to avoid selection bias and to allow complete diagnostic accuracy analyses. The pilot publication reported feasibility and qualitative test characteristics only; no quantitative analyses, threshold-based classification, or diagnostic accuracy measures presented in the current study were part of the earlier report.

### 2.2 Patient enrollment

Consecutive patients presenting with acute ischemic stroke (AIS) or transient ischemic attack were screened for eligibility over a 12 months period (June 2023 to May 2024). Inclusion criteria required patients to have (1) confirmed DOAC intake within the previous 24 hours or no DOAC use within the past 7 days, as verified by clinical history and patient records, and (2) the ability to provide blood and urine samples. Exclusion criteria included severe renal impairment (estimated glomerular filtration rate < 30 mL/min/1.73m^2^), inability to provide informed consent, or insufficient sample volumes for testing. Transient ischemic attack was defined as a focal neurological deficit lasting < 24 hours, whereas ischemic stroke was defined as a persisting deficit ≥24 hours in the absence of alternative causes. All patients underwent non-contrast computed tomography (CT) on admission to exclude intracranial hemorrhage. The presence of an ischemic parenchymal lesion on CT or CT-perfusion imaging was not required for study inclusion.

### 2.3 Urine-based POC testing

All participants underwent index testing using the DOASENSE (*DOASENSE GmbH*) urine dipstick. The dipstick contains separate reaction pads for factor-Xa inhibitors (FXaIs) and dabigatran and was applied according to the manufacturer’s instructions. Test results were recorded as both visual readouts (negative, +, or ++) and automatic digital readouts from the DOASENSE reader. Treating clinicians and laboratory staff performing reference assays were blinded to each other’s results [17,20].

### 2.4 General data acquisition

Clinical data were collected, including age, sex, medical history (e.g., atrial fibrillation and renal impairment), stroke severity as measured by the National Institutes of Health Stroke Scale, and prior anticoagulation use. Laboratory evaluations included blood-based coagulation parameters, such as international normalized ratio and activated partial thromboplastin time. Plasma levels of FXa inhibitors (apixaban, rivaroxaban, and edoxaban) were measured using drug-specific chromogenic anti-Xa assays with appropriate calibrators. Dabigatran activity was quantified using the Biophen DTI assay with drug-specific calibrators. All assays were performed on a fully automated coagulation analyzer operated by the hospital’s 24/7 emergency coagulation service.

Two prespecified thresholds for diagnostic classification were applied:

1. Relevant anticoagulant activity: anti-Xa ≥ 30 ng/mL
2. IVT-relevant threshold: anti-Xa < 100 ng/mL, reflecting emerging observational evidence that IVT may be considered below this level [18].

Due to the small sample size, no separate dabigatran-specific performance analysis was undertaken.

### 2.5 Reporting guideline

The Standards for Reporting Diagnostic Accuracy Studies checklist is provided in Supplementary Table S1.

### 2.6 Sample size and precision

This study used a prospective consecutive cohort over a predefined inclusion period. No formal power calculation was performed due to uncertain on-therapy prevalence; instead, diagnostic precision was quantified using exact 95% CIs at the predefined thresholds (≥30 ng/mL and ≥100 ng/mL). The pragmatic sample size and its effect on CI width—particularly in low-prevalence subgroups such as dabigatran—are acknowledged in the limitations.

### 2.7 Handling of missing data

Primary accuracy analyses followed a complete-case approach requiring paired index test and reference standard results. Cases with missing reference or urine samples were excluded from accuracy calculations but retained in descriptive summaries. The number of patients contributing to each analysis is reported in the Tables and Results section.

### 2.8 Outcomes

The primary endpoint was the sensitivity of the urine-based dipstick test for detecting specific DOAC levels ≥ 30 ng/mL. Secondary endpoints included specificity, diagnostic accuracy for identifying patients with specific DOAC levels ≥ 100 ng/mL (a clinically relevant threshold for thrombolysis), and time efficiency (i.e., time from sampling to result) compared to standard blood-based testing.

### 2.9 Statistical Analysis

Descriptive statistics were presented as median (interquartile range) or proportions, as appropriate. Diagnostic performance metrics, including sensitivity, specificity, and accuracy, were calculated with 95% CIs. For diagnostic proportions (sensitivity, specificity, positive predictive value, and negative predictice value), 95% CIs were calculated using the exact binomial (Clopper–Pearson) method. Likelihood ratios (LR+, LR−) are presented with 95% CIs derived via the log (Katz) method; when a contingency-table cell contained zero, a Haldane–Anscombe correction (0.5) was applied prior to calculation. ROC AUC is reported with nonparametric DeLong 95% CIs as provided by SPSS. All CIs are 2-sided with α = 0.05. Receiver operating characteristic (ROC) analysis assessed dipstick performance at predefined anti-Xa thresholds. Time-to-result comparisons between urine-based and blood-based testing were evaluated using nonparametric methods. Statistical significance was set at *P* < .05, with analyses performed using IBM SPSS Statistics 22.0 (IBM; https://www.ibm.com/de-de/products/spss-statistics).

## 3 Results

### 3.1 Participant flow

**Figure 1:**
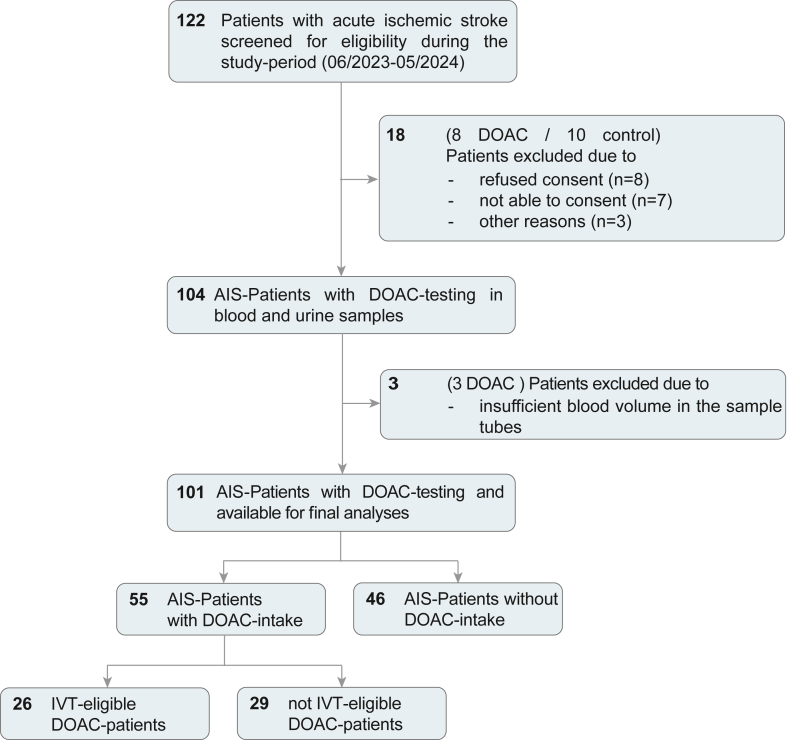
Flowchart of included patients. This flowchart summarizes patient screening and inclusion during the study (June 2023 to May 2024). Of 122 acute ischemic stroke patients screened, 21 were excluded due to consent or sample issues. The final cohort included 101 patients: 55 with and 46 without DOAC intake, undergoing both blood and urine testing. 26 of 55 DOAC patients were rated as IVT-eligible due to drug-specific DOAC levels equal or below 100 ng/mL. DOAC, direct oral anticoagulant.

The flow of is shown in the Figure 1. A total of 122 patients with AIS were screened for eligibility, of whom 18 were excluded due to refusal or inability to consent. After excluding an additional 3 patients due to insufficient blood volume, the final analysis included 101 AIS patients, comprising 55 patients with DOAC intake and 46 controls.

### 3.2 Baseline characteristics

Patients in the DOAC group had a lower median age compared to the control group (64 years [IQR: 56-76] versus 81 years [IQR: 73-85]) and exhibited a higher prevalence of atrial fibrillation (85.5% vs 8.7%). Stroke severity at admission, assessed using the National Institutes of Health Stroke Scale, was similar between the groups, with a median score of 2 (IQR: 0-5) in the DOAC group and 2 (IQR: 0-4) in the control group. Intravenous thrombolysis was performed less frequently in DOAC patients (16.4%) than in controls (32.6%). Other baseline characteristics, including premorbid disability, vascular risk factors, and comorbidities, are detailed in Table 1.

**Table 1:** Baseline and laboratory characteristics of included patients.

Patients with AIS (*N* = 101)	DOAC (*n* = 55)	Control (*n* = 46)
Age (y), median (IQR)	64 (56-76)	81 (73-85)
Female sex; *n* (%)	26 (47.3%)	17 (37.0%)
Prior medical history; *n* (%)		
Premorbid mRS; median (IQR)	1 (0-2)	0 (0-1)
Arterial hypertension	44 (80.0%)	28 (60.9%)
Peripheral arterial occlusive disease	4 (7.3%)	3 (6.5%)
Coronary artery disease	14 (25.5%)	2 (4.3%)
Atrial fibrillation	47 (85.5%)	4 (8.7%)
Renal impairment	9 (16.4%)	1 (2.2%)
Prior stroke or TIA	15 (27.3%)	5 (10.9%)
Body weight, kg; median (IQR)	82 (75-91)	82 (70-89)
Comedication; *n* (%)		
Platelet function inhibitor	4 (7.3%)	43 (93.5%)
Statin	52 (94.5%)	44 (95.7%)
Stroke characteristics		
Acute ischemic stroke; *n* (%)	38 (69.1%)	34 (73.9%)
Transient ischemic attack; *n* (%)	17 (30.9%)	12 (26.1%)
NIHSS on admission; median (IQR)	2 (0-5)	2 (0-4)
Mild (NIHSS 0-4); *n* (%)	41 (74.5%)	36 (78.3%)
Intravenous thrombolysis; *n* (%)	9 (16.4%)	15 (32.6%)
Endovascular thrombectomy; *n* (%)	10 (18.2%)	4 (8.7%)
Length of hospital stay, d; median (IQR)	8 (5-13)	5 (4-8)
mRS at discharge; median (IQR)	2 (1-3)	1 (0-2)
DOAC agent, *n* (%)		
Rivaroxaban	7 (12.7%)	**-**
Apixaban	44 (80.0%)	**-**
Edoxaban	2 (3.6%)	**-**
Dabigatran	2 (3.6%)	**-**
Reduced dose	18 (32.7%)	**-**
Last intake of DOAC, h; median (IQR)	3 (2-6)	**-**
Within the last 3 h; *n* (%)	18 (50.9%)	**-**
Duration of observed DOAC intake, d; median (IQR)	2 (1-2)	**-**
Indication for OAC; *n* (%)		
Atrial fibrillation	47 (85.5%)	**-**
Deep vein thrombosis	0 (0)	**-**
Pulmonary embolism	1 (1.8%)	**-**
Other	5 (9.1%)	**-**
Laboratory measurements, median (IQR)		
INR	1.1 (1.0-1.1)	1.0 (1.0-1.1)
aPTT, s	33 (29-37)	28 (26-31)
Quick value, %	87 (78-97)	98 (89-106)
Thrombocyte count, 10∗9/L	233 (174-271)	245 (199-271)
Creatinine, mg/dL	0.9 (0.7-1.2)	0.8 (0.7-1.0)
Estimated GFR, mL/min/1.73m^2^	75 (58-101)	91 (79-108)
Urea, mg/dL	29 (25-40)	27 (21-35)
Specific anti-Xa levels (fXi-users only)		
Specific anti-Xa activity, ng/mL; median (IQR)	110 (60-190)	0 (0-0)
Specific Anti-Xa activity ≥ 30 ng/mL; *n* (%)	47/53 (88.7)	0 (0%)
Specific anti-Xa activity ≥ 100 ng/mL; *n* (%)	32/53 (60.4)	0 (0%)
Dipstick results; *n* (%)		
Automatic result positive	49 (89.1%)	0 (0%)
Visual result		
Unsuitable sample according to urine color	0 (0%)	0 (0%)
Low creatinine	1 (1.8%)	2 (4.3%)
Factor-Xa inhibitor negative	5 (9.1%)	46 (100%)
Factor-Xa inhibitor +	18 (32.7%)	0 (0%)
Factor-Xa inhibitor ++	32 (58.2%)	0 (0%)
Time intervals		
Blood sample to result, min; median (IQR)	144 (78-339)	161 (108-333)
Urine sample to result, min; median (IQR)	19 (15-27)	17 (15-29)

Provided are the baseline characteristics of patients dichotomized into DOAC intake and control group. Data are presented as median (interquartile’s range) or absolute number (percentage). For patients with DOAC intake only (*n* = 55), information about DOAC agent, last intake, dosing and indication for oral anticoagulation are shown. Reduced dose was defined as daily dose of less than 20 mg rivaroxaban, 10 mg apixaban, or 60 mg edoxaban.

aPTT, activated partial thromboplastin time; d, days; DOAC, direct oral anticoagulant; GFR, glomerular filtration rate; h, hours; IQR, interquartile’s range; INR, international normalized ratio; SD, standard deviation; y, years; mRS, modified Rankin scale (ranging from 0, no symptoms, to 6, dead); NIHSS, National Institute of Health Stroke Scale (ranging from 0 to 42 with higher scores indicating more severe symptoms).

### 3.3 DOAC Characteristics

**Figure 2:**
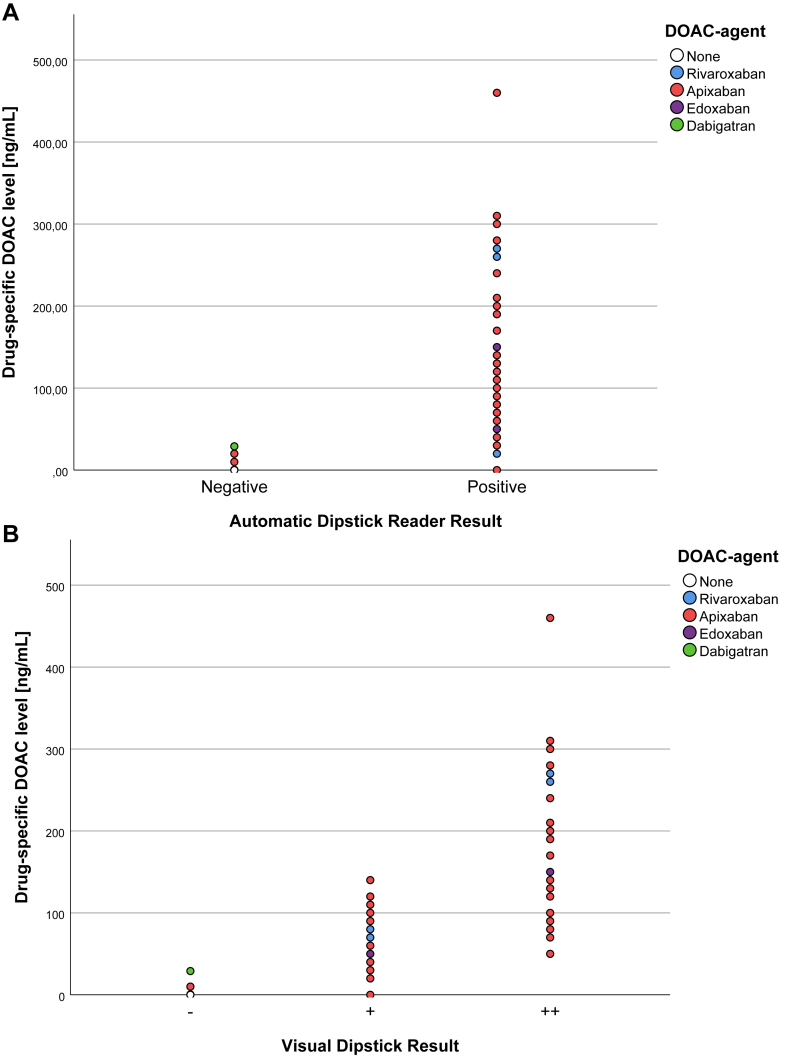
Distribution of apixaban and rivaroxaban anti-Xa levels and dabigatran activity measured by ecarin-clotting time according to: (A) automatic dipstick result and (B) visual dipstick result. Panels A and B illustrate drug-specific DOAC levels stratified by the automatic (negative and positive) and visual dipstick test results (−, +, ++). Patients with DOAC intake are represented by colored circles, while control group data are shown as white circles.

Among patients with DOAC intake (*n* = 55), apixaban was the most frequently used agent (80.0%), followed by rivaroxaban (12.7%), edoxaban (3.6%), and dabigatran (3.6%). A reduced DOAC dose was administered to 32.7% of patients. The median time since last DOAC intake was 3 hours (IQR: 2-6), with 50.9% of patients having taken their last dose within the preceding 3 hours. The primary indication for DOAC therapy was atrial fibrillation (85.5%). The distribution of anticoagulant activity by drug class and the decision thresholds applied in our analyses are shown in Figure 2. Only 2 patients with dabigatran-intake were present in our cohort both were negative on ecarin-clotting-time (ECT) testing and had a negative POC-testing result. Therefore, they were excluded for further analyses of in factor Xa inhibitor patients. The median anti-Xa activity in the DOAC group was 110 ng/mL (IQR: 60-190), with 88.7% of patients exhibiting anti-Xa levels ≥ 30 ng/mL and 60.4% levels ≥100 ng/mL, the latter classified as noneligible for IVT. In contrast, no measurable anti-Xa activity was detected in the control group (Table 1).

### 3.4 Time to Result

Median time from sample collection to result was 19 minutes (IQR: 15-27) for the urine dipstick test and 144 minutes (IQR: 78-339) for blood-based specific anti-Xa testing. This represents a median time difference of 125 minutes (Table 1).

### 3.5 Diagnostic performance for relevant DOAC activity

The urine-based DOAC dipstick demonstrated high diagnostic accuracy in our sample (Table 2). The automatic readout exhibited a sensitivity of 100% and specificity of 96.3% for detecting anti-Xa activity ≥ 30 ng/mL. Similarly, visual interpretation of the dipstick (+ / ++) yielded a sensitivity of 100% and specificity of 94.4% for detecting anti-Xa activity ≥ 30 ng/mL.

**Table 2:** Sensitivity and specificity analyses for different testing thresholds.

Overall automatic (*N* = 101)	True positive/condition positive	True negative/condition negative	Sensitivity (95% CI)	Specificity (95% CI)
Cutoff ≥30	47/47	52/54	100.0% (92.4%-100%)	96.3% (87.3%-99.6%)
Cutoff ≥100	32/32	52/69	100% (89.1%-100%)	75.4% (63.5%-85.0%)
**Overall– visual + / ++ (*N* = 101)**				
Cutoff ≥30	47/47	51/54	100% (92.5%-100%)	94.4% (84.6%-98.8%)
Cutoff ≥100	32/32	51/69	100% (89.1%-100%)	73.9% (61.9%-83.8%)
**Overall– visual ++ (*N* = 101)**				
Cutoff ≥30	32/47	54/54	68.1% (52.9%-80.9%)	100% (93.4%-100%)
Cutoff ≥100	27/32	64/69	84.4% (67.2%-94.7%)	92.8% (83.9%-97.6%)

The table presents diagnostic accuracy metrics for three dipstick readouts—automatic positive, visual positive (+/++), and visual double-positive (++). Sensitivity and specificity are reported for detecting plasma DOAC activity above two predefined thresholds: ≥30 ng/mL (standard screening threshold) and ≥100 ng/mL (IVT-relevant threshold). True positive/condition positive indicates the proportion of true positives among all patients whose plasma DOAC level exceeded the respective threshold. True negative/condition negative indicates the proportion of true negatives among all patients whose plasma DOAC level remained below the threshold. Exact 95% CI are shown for sensitivity and specificity.

### 3.6 Diagnostic performance for identification of IVT-ineligible patients with fXa-inhibitors

To assess the clinical applicability of the dipstick for identifying patients unlikely to be eligible for IVT, diagnostic accuracy was evaluated at the IVT-relevant threshold of anti-Xa < 100 ng/mL (Table 2). For the automatic readout, sensitivity was 100% (32/32), correctly identifying all patients with anti-Xa ≥ 100 ng/mL, while specificity was 75.4% (52/69), reflecting the reclassification of 17 patients with anti-Xa < 100 ng/mL as false positives. A similar pattern was observed for the visual +/++ readout (sensitivity 100% [32/32] and specificity 73.9% [51/69]). The accuracy of the automatic readout was 98.0% (98/101) for the ≥ 30 ng/mL threshold and 83.2% (83/101) for the ≥ 100 ng/mL threshold, whereas the visual (+/++) readout achieved accuracies of 97.0% (98/101) and 82.2% (83/101), respectively.

In contrast, the stricter visual ++ criterion demonstrated higher specificity (92.8%, 64/69) with preserved overall discriminatory ability (sensitivity 84.4%, 27/32). This readout substantially reduced the number of false positives—misclassifying only 5 of the 69 patients with anti-Xa < 100 ng/mL—while missing 5 of the 32 patients above the threshold (false negatives). The overall accuracy for this threshold was 90.1% (91/101; Table 2).

These findings illustrate the inherent trade-off between sensitivity and specificity across dipstick readout modalities. Both the automatic and visual +/++ classifications maximized safety by identifying all patients with elevated DOAC levels, thereby minimizing the risk of inappropriate IVT administration. Conversely, the visual ++ strategy offered greater specificity and fewer unnecessary exclusions of potentially IVT-eligible patients, albeit at the cost of reduced sensitivity. The choice of operational threshold may therefore depend on local priorities—maximal safety versus maximal preservation of IVT eligibility—and warrants prospective evaluation in real-world clinical workflows.

### 3.7 ROC-analysis of association of Anti-Xa levels with Dipstick results

**Figure 3:**
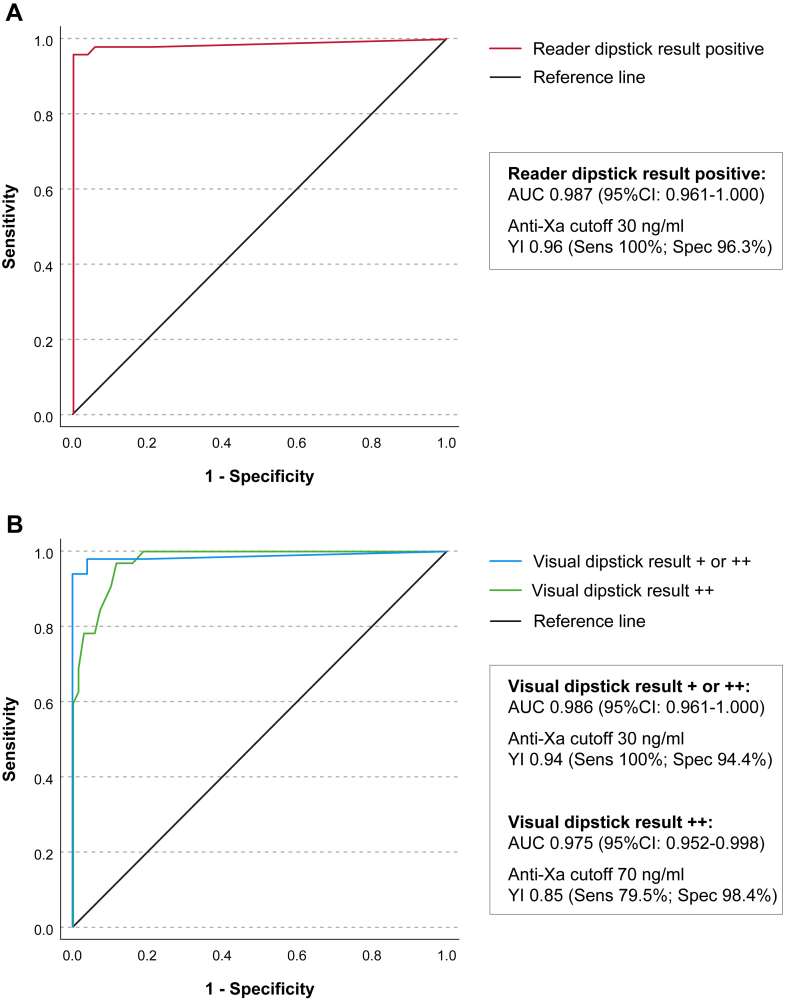
Receiver-operating-characteristic analysis relating drug-specific anti-Xa levels to (A) automated dipstick reader results and (B) visual dipstick classifications. Analysis was performed in patients with factor Xa-inhibitor intake only. The optimal anti-Xa cut-off for detecting any DOAC activity was 30 ng/mL for both visual (+/++) and automated readouts (AUC 0.986-0.987, YI 0.94-0.96). For strong visual positivity (++), the optimal discriminatory threshold was 70 ng/mL (AUC 0.975, sensitivity 79.5%, and specificity 98.4%). AUC, area under the curve; YI, Youden index.

ROC analyses identified an optimal anti-Xa cut-off of 30 ng/mL for detecting any relevant anticoagulant activity using both the automated (Figure 3 A) and the visual (either + or ++) dipstick readouts (Figure 3 B), yielding AUC values of 0.986 to 0.987 and Youden indices of 0.94 to 0.96. In contrast, the stricter visual category (++) demonstrated its best discriminatory performance at a higher cut-off of 70 ng/mL, corresponding to an AUC of 0.975, a Youden index of 0.85, a sensitivity of 79.5%, and a specificity of 98.4% (Figure 3 B).

## 4 Discussion

The urine-based DOAC dipstick demonstrated high sensitivity and specificity for detecting relevant anticoagulatory activity (≥30 ng/mL) as well as for identifying patients within the clinically meaningful threshold for thrombolysis eligibility (≤100 ng/mL) in this cohort. Both the visual and automatic readouts achieved robust diagnostic performance, with accuracy values of up to 90% for the stricter high-level visual criterion, underscoring the reliability of the test in routine clinical decision-making. Moreover, the semi-quantitative visual categories provided clinically meaningful stratification of anti-Xa levels, closely mirroring the distribution observed in blood-based assays.

The significant reduction in time-to-result for urine-based testing compared to blood-based specific anti-Xa assays underscores its potential to address time constraints in AIS management. The median time savings of over 2 hours may be particularly relevant in scenarios where rapid decision-making is required, such as evaluating thrombolysis eligibility [21,22].

According to current ESO guidelines, IVT remains contraindicated in patients with relevant DOAC activity due to bleeding risk [10]; however, emerging observational data suggest that thrombolysis may be safe in selected patients even with anti-Xa levels up to 100 ng/mL [14,23,24]. In this context, our findings support the use of visual +/++ or automatic dipstick readouts to ensure maximal safety, as both reliably identified all patients ≥ 100 ng/mL. Yet, this approach excluded a substantial number of patients with lower DOAC levels who may have been IVT-eligible. The visual ++ criterion, while slightly less sensitive, offered greater specificity and preserved more candidates for treatment. This trade-off may be acceptable in clinical settings aiming to balance safety with timely recanalization [21], particularly where rapid quantitative testing is unavailable [25,26].

Several limitations merit consideration. This was a single center study with a pragmatic, consecutive enrollment strategy, which may limit generalizability and precision, particularly in low-prevalence subgroups. The sample size was adequate for initial accuracy estimation but requires confirmation in larger multicenter cohorts. Feasibility of urine collection in hyperacute stroke—especially in patients unable to provide spontaneous samples—was not systematically assessed. Dabigatran-treated patients were scarce (*n* = 2), precluding ROC or threshold analyses for this drug class; consequently, performance estimates primarily reflect FXa inhibitors. Nonetheless, we report dabigatran activity using ECT and qualitative dipstick detection to reflect real-world workflows in which identification of dabigatran may guide idarucizumab reversal prior to IVT.

In settings without immediate access to DOAC-specific assays, dabigatran can be screened using TT, dTT, or ecarin-based assays, whereas normal PT/international normalized ratio or activated partial thromboplastin time should not be used to exclude clinically meaningful DOAC activity; calibrated anti-Xa remains preferred for FXa inhibitors. Our center operated with continuous 24/7 availability of calibrated assays, and all diagnostic estimates presented derive exclusively from these drug-specific measurements. Finally, we adopted the Standards for Reporting Diagnostic Accuracy Studies framework to enhance transparency and explicitly report sample size rationale, missing-data handling, and exact 95% CIs to avoid overinterpretation of estimates with limited precision.

## 5 Conclusion

The urine-based DOAC dipstick offers a rapid and reliable method for assessing relevant anticoagulatory activity in AIS patients, particularly at thresholds critical for thrombolysis decision-making. Future multicenter studies are needed to validate these findings and explore the test’s integration into acute stroke care protocols.
