# Supplementary material for: Urine-based point-of-care detection of direct oral anticoagulant activity in acute stroke—accuracy at anti-Xa thresholds relevant for intravenous thrombolysis
Source: Res Pract Thromb Haemost. 2025 Dec 29;10(1):103331. doi: 10.1016/j.rpth.2025.103331 (PMC12860936; doi:10.1016/j.rpth.2025.103331)
Supplement: Supplementary Table S1 [file mmc1.docx]

**Supplementary Tables and Figures:**

**Supplementary Table S1. STARD 2015 checklist for reporting diagnostic accuracy studies (updated to current manuscript)**

| Section | STARD item | Item description (abridged) | Where reported (section/figure/table) |
| --- | --- | --- | --- |
| Title/Abstract | 1 | Identification as a diagnostic accuracy study | Title; Abstract |
| Title/Abstract | 2 | Structured summary incl. index test, reference standard, participants, accuracy estimates, timing | Abstract |
| Introduction | 3 | Scientific and clinical background | Introduction (DOAC challenges in IVT; need for rapid testing) |
| Introduction | 4 | Study objectives and hypotheses | Introduction (last paragraph: aims to evaluate sensitivity/specificity/time-efficiency; thresholds 30 & 100 ng/mL) |
| Methods—Participants | 5 | Study design (prospective/observational) | Methods → Study Design and Setting |
| Methods—Participants | 6 | Eligibility criteria | Methods → Patient Enrollment (inclusion/exclusion) |
| Methods—Participants | 7 | Setting, locations, dates | Methods → Study Design and Setting; Patient Enrollment (06/2023–05/2024) |
| Methods—Participants | 8 | Participant series (consecutive/random) | Methods → Patient Enrollment (consecutive screening) |
| Methods—Test Methods | 9 | Index test details (replicable description) | Methods → Data Acquisition (Doasense™ dipstick; automatic & visual readouts; thresholds) |
| Methods—Test Methods | 10 | Reference standard details | Methods → Data Acquisition (drug-specific anti-Xa; ECT for dabigatran) |
| Methods—Test Methods | 11 | Rationale for reference standard | Introduction (threshold rationale 30 & 100 ng/mL); Methods → Data Acquisition |
| Methods—Test Methods | 12a | Definition & rationale of index-test positivity cut-offs | Introduction; Methods → Outcomes |
| Methods—Test Methods | 12b | Definition & rationale of reference-standard cut-offs | Introduction; Methods → Outcomes |
| Methods—Bias | 13 | Blinding of index test performers/readers to reference standard | Methods → (state blinding if applicable) / Statistical Analysis |
| Methods—Bias | 14 | Blinding of reference-standard assessors to index test | Methods → (state blinding if applicable) / Statistical Analysis |
| Methods—Analysis | 15 | Handling of indeterminate results | Methods → Statistical Analysis; Table 2 footnotes (if any indeterminate) |
| Methods—Analysis | 16 | Handling of missing data | Methods → Handling of missing data; Results → Denominators and missingness; |
| Methods—Analysis | 17 | Analysis of variability (pre-specified vs exploratory) | Methods → Statistical Analysis (predefined thresholds; subgroup consistency) |
| Methods—Analysis | 18 | Intended sample size and how it was determined | Methods → Sample size and precision |
| Results—Participants | 19 | Flow of participants | Results → Participant flow; Figure 1 |
| Results—Participants | 20 | Baseline characteristics | Results → Baseline Characteristics; Table 1 |
| Results—Participants | 21a | Distribution of disease severity (if applicable) | Results → NIHSS baseline (Table 1) |
| Results—Participants | 21b | Alternative diagnoses (if applicable) | Not applicable |
| Results—Test Results | 22 | Time interval & any interventions between index and reference | Methods → Data Acquisition/Workflow; Results → Time to Result |
| Results—Test Results | 23 | Cross-tabulation of index vs reference standard | Table 2 (true positive/true negative; denominators labeled) |
| Results—Accuracy | 24 | Estimates of accuracy and 95% CIs | Results → Diagnostic Performance (≥30 & ≥100 ng/mL); Table 2 |
| Results—Harms | 25 | Adverse events from testing | Not observed/Not applicable; see Discussion if needed |
| Discussion | 26 | Study limitations, sources of bias, generalisability | Discussion (single-centre; sample size; feasibility; dabigatran n=2; precision) |
| Discussion | 27 | Implications for practice (intended use, clinical role) | Discussion (IVT decision-making; safety/eligibility trade-offs; settings without quantitative testing) |
| Other | 28 | Registration | Methods → Study Design and Setting (NCT06037200) |
| Other | 29 | Protocol access | On request |
| Other | 30 | Funding/role of funders | Funding section (DOASENSE device support; no influence) |

Caption: STARD checklist updated to reflect the latest manuscript sections, figures, and tables.
